# IGFBP7 promotes endothelial cell repair in the recovery phase of acute lung injury

**DOI:** 10.1042/CS20240179

**Published:** 2024-06-21

**Authors:** Rui He, Bo Feng, Yuezhou Zhang, Yuqing Li, Daoxing Wang, Linchao Yu

**Affiliations:** 1 Department of Respiratory Medicine, the Second Affiliated Hospital of Chongqing Medical University, Chongqing, China; 2 Department of Respiratory Medicine, People’s Hospital of Tongnan District, Chongqing, China; 3 Department of Hepatobiliary Surgery, The Second Affiliated Hospital of Chongqing Medical University, Chongqing, China; 4 Chongqing Health Commission Key Laboratory for Respiratory Inflammation Damage and Precision Medicine

**Keywords:** ALI, c-fos, cell proliferation, igfbp7, yap

## Abstract

IGFBP7 has been found to play an important role in inflammatory diseases, such as acute lung injury (ALI). However, the role of IGFBP7 in different stages of inflammation remains unclear. Transcriptome sequencing was used to identify the regulatory genes of IGFBP7, and endothelial IGFBP7 expression was knocked down using Aplnr-Dre mice to evaluate the endothelial proliferation capacity. The expression of proliferation-related genes was detected by Western blotting and RT-PCR assays. In the present study, we found that knockdown of IGFBP7 in endothelial cells significantly decreases the expression of endothelial cell proliferation-related genes and cell number in the recovery phase but not in the acute phase of ALI. Mechanistically, using bulk-RNA sequencing and CO-IP, we found that IGFBP7 promotes phosphorylation of FOS and subsequently up-regulates YAP1 molecules, thereby promoting endothelial cell proliferation. This study indicated that IGFBP7 has diverse roles in different stages of ALI, which extends the understanding of IGFBP7 in different stages of ALI and suggests that IGFBP7 as a potential therapeutic target in ALI needs to take into account the period specificity of ALI.

## Introduction

Acute lung injury (ALI) is a life-threatening disease with a high mortality rate. ALI is divided into three stages: the acute exudative phase, the fibroproliferative phase, and the recovery phase. The acute exudative phase, occurring in the early stage of ALI, is characterized by increased permeability due to damage to the alveolar-capillary membrane. The fibroproliferative phase is characterized by infiltration of acute and chronic inflammation, proliferation of fibroblasts, and deposition of collagen. The recovery phase involves the repair of the microvascular endothelial barrier [1,2]. The pulmonary microvasculature is the main structure damaged by the acute inflammatory response during ALI [3], which leads to microvascular barrier dysfunction. In contrast, during the recovery stage of inflammation, proliferation of the microvascular endothelium promotes the repair of endothelial barrier function. However, the mechanisms of microvascular endothelial repair during the inflammation remain unclear.

Insulin-like growth factor binding protein 7 (IGFBP7), also known as angiomodulin, is a secreted protein that couples to IGF1R, blocks IGF activity [4], and is expressed in various endothelial and epithelial cells [5]. Unlike other IGFBPs, IGFBP7 has more IGF-independent functions since IGFBP7 possesses a lower affinity for IGF. In the clinic, IGFBP7 is a specific biomarker for acute kidney injury [6] and has also been found to exacerbate renal tubular injury [7]. IGFBP7 promotes programmed cell death and inflammatory responses through the IGF1R receptor [8]. In addition, IGFBP7 also regulates the proliferation and migration of cells [9]. Studies have found IGFBP7 prevents the senescence of dental pulp-derived mesenchymal stem cells and promotes tissue regeneration [10]. IGFBP7 has been found to promote the proliferation and migration of vascular endothelial cells in tumor vasculature and, therefore, is expected to be a potential target for tumor therapy [11–13]. For example, several research teams have reported that IGFBP7 is highly expressed in tumor cells [14], thereby promoting tumor angiogenesis [11], and that blockade of IGFBP7 significantly impairs tumor vascular remodeling [15]. These studies suggest that IGFBP7 plays diverse roles in different diseases. Our previous research has indicated that IGFBP7 exacerbates inflammation-induced endothelial injury during the acute phase of ALI [16]. However, the role of IGFBP7 in endothelial barrier function during the recovery phase of ALI remains unclear.

Yes-associated protein 1 (YAP1) is a crucial downstream effector molecule of the Hippo pathway, which plays an essential role in regulating organ development and cell proliferation [17]. When the Hippo pathway is activated, a series of kinase cascades lead to phosphorylation of the YAP1 molecule and retention in the cytoplasm, which inhibits downstream gene expression. In contrast, dephosphorylation of YAP1 enters the nucleus and promotes the expression of genes downstream of transcription [18]. Previous studies have reported that YAP1 expression is dysregulated in endothelial cells of ARDS (acute respiratory distress syndrome) and that YAP1 overexpression protects against endothelial damage caused by LPS [19]. However, the interaction of IGFBP7 with YAP1 phosphorylation in ALI is still poorly understood.

In this study, we found that the knockdown of IGFBP7 in endothelial cells significantly decreases the expression of endothelial cell proliferation-related genes and cell number in the recovery phase but not in the acute phase of ALI. Mechanistically, using bulk-RNA sequencing and CO-IP, we found that IGFBP7 promotes phosphorylation of FOS and subsequently up-regulates YAP1 molecules, thereby promoting endothelial cell proliferation. The present study found that IGFBP7 has diverse roles in different stages of ALI, which broaden the understanding of IGFBP7 at different stages of ALI.

## Results

### IGFBP7 promotes endothelial cell proliferation and repair

**Figure 1:**
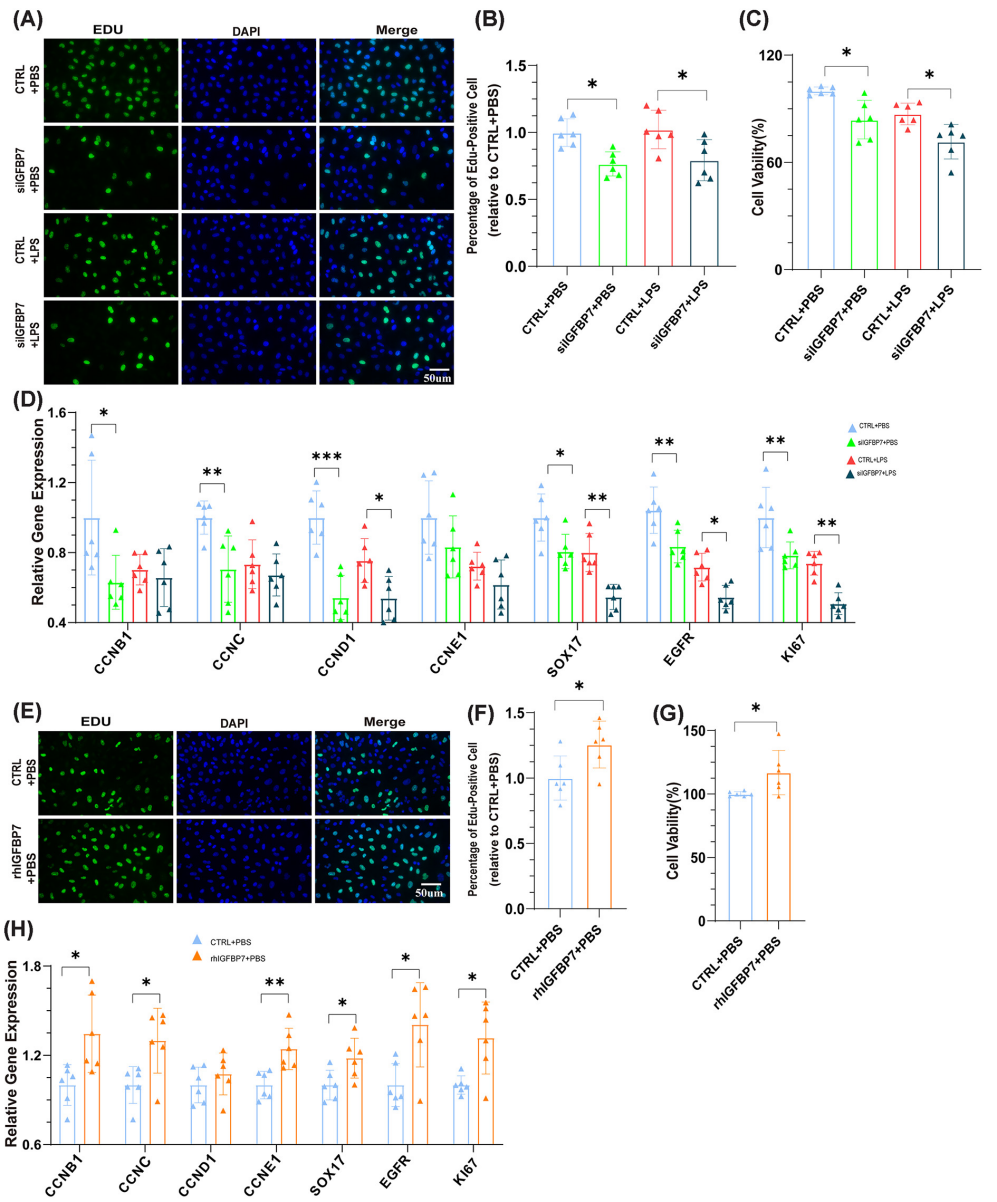
IGFBP7 promotes HUVECs proliferation (**A,B**) Representative images of EDU-AF488 and DAPI co-staining in HUVECs transfected with IGFBP7 siRNA (48 h) and treated with LPS (5 µg/ml) for 24 h. Scale bars: 50 μm (40×). (**C**) Determined by the CCK-8 assay, the cell viability of HUVECs transfected with IGFBP7 siRNA for 48 h and treated with LPS (5 µg/ml) for 24 h. (**D**) Assessed by qRT-PCR, the mRNA expression of CCNB1, CCNC, CCND1, CCNE1, SOX17, EGFR, and KI67 in HUVECs transfected with IGFBP7 siRNA for 48 h and treated with LPS (5 µg/ml) for 24 h. (**E,F**) Confocal microscopy images depicting EDU-AF488 staining alongside DAPI staining in healthy HUVECs intervened with rhIGFBP7 (1 μg/ml) for 48 h. Scale bars: 50 μm (40×). (**G**) Determined by the CCK-8 assay, the cell viability of HUVECs intervened with rhIGFBP7 (1 μg/ml) for 48 h. (**H**) Assessed by qRT-PCR, the mRNA expression of CCNB1, CCNC, CCND1, CCNE1, SOX17, EGFR, and KI67 in HUVECs intervened with rhIGFBP7 (1 μg/ml) for 48 h. Data represented as means ± SDs. *P<0.05, **P<0.01, ***P<0.005 [one-way ANOVA, Tukey’s test (B–D) and *t*-test (F**–**H)].

**Figure 2:**
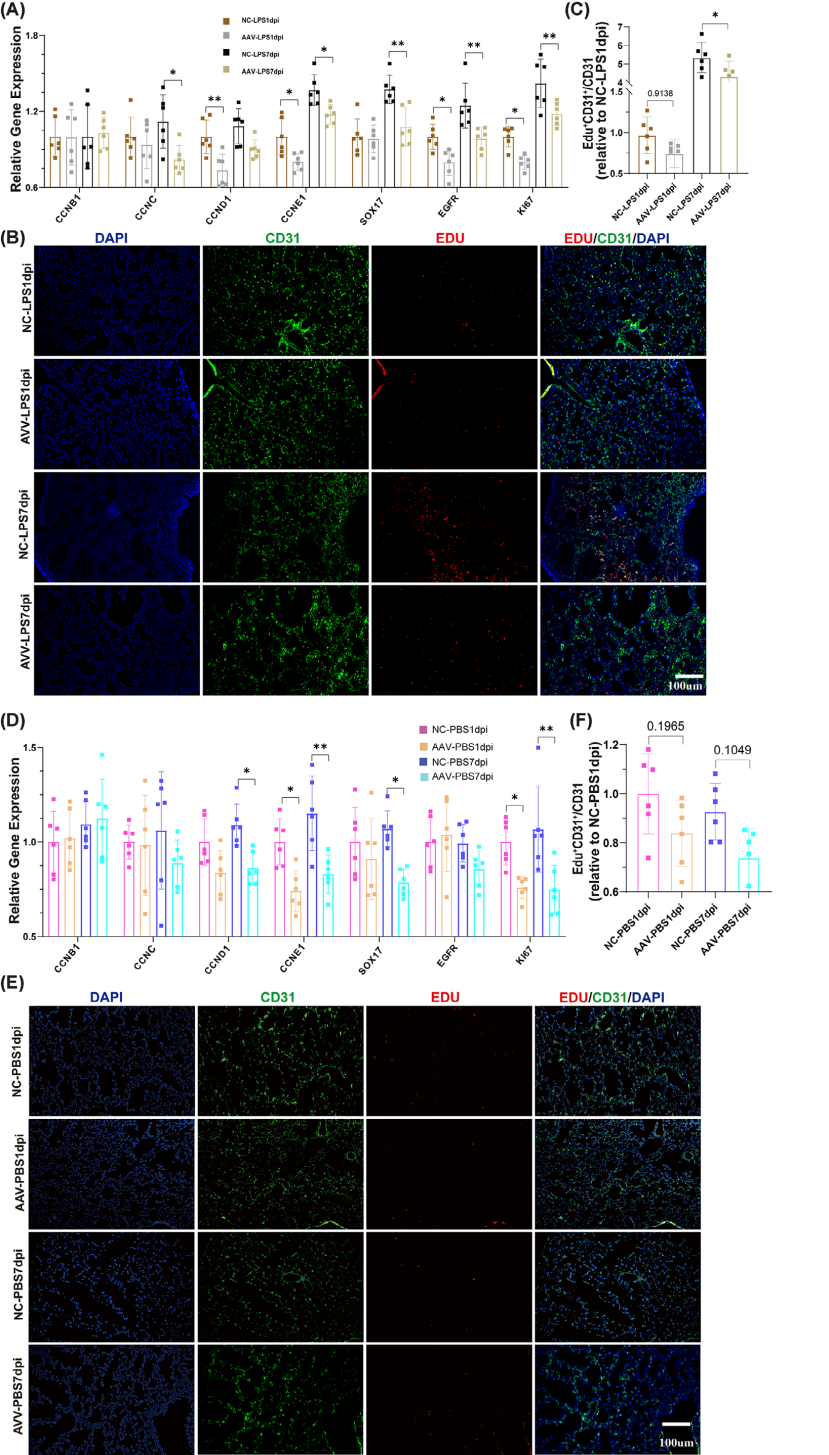
The knockdown of IGFBP7 reduced vascular endothelial cell proliferation in healthy and ALI mice (**A**) Assessed by qRT-PCR, the mRNA expression of CCNB1, CCNC, CCND1, CCNE1, SOX17, EGFR, and KI67 in mouse lung tissues from mice subjected to intratracheal injection with LPS (5 mg/kg) treatment and collecting lung tissues after 24 h or 7 days. (**B,C**) Representative images of EDU-AF555, CD31-AF488, and DAPI co-staining in mouse lung tissues from mice subjected to intratracheal injection with saline or LPS (5 mg/kg) treatment and collecting lung tissues after 24 h or 7 days. Scale bars: 100 μm (20×). (**D**) Assessed by qRT-PCR, the mRNA expression of CCNB1, CCNC, CCND1, CCNE1, SOX17, EGFR, and KI67 in mouse lung tissues from mice subjected to intratracheal injection with saline treatment and Collecting lung tissues after 24 h or 7 days. (**E,F**) Representative images of EDU-AF555, CD31-AF488, and DAPI co-staining in mouse lung tissues from mice subjected to intratracheal injection with saline treatment and Collecting lung tissues after 24 h or 7 days. Scale bars: 100 μm (20×). Data represented as means ± SDs. *P<0.05, **P<0.01 [one-way ANOVA, Tukey’s test (A,C,D,F)]. AAV, Adeno-associated virus; NC, Negative Control.

Previous studies have reported that genes such as Ki67 [20] /Egfr [21] /Sox17 [22] /Cyclins [23] are actively expressed in proliferating cells, indicating that the cells are in an active state of proliferation. To investigate the role of IGFBP7 on endothelial cell growth, we first examined the role of IGFBP7 in the injured endothelial model (LPS stimulation). We found that the knockdown of IGFBP7 reduced the activation of nuclear genes (Figure 1A,B), inhibited cell proliferation (Figure 1C), and suppressed the expression of cell proliferation-related genes (Figure 1D) in injured endothelial. We then perturbed endothelial cell IGFBP7 and analyzed changes in healthy endothelial cells. We found that the knockdown of IGFBP7 still down-regulated the activation of nuclear genes, inhibited cell proliferation (Figure 1A–C), and suppressed the expression of cell proliferation-related genes in endothelial cells (Figure 1D). Moreover, recombinant human IGFBP7 (rhIGFBP7) promoted the activation of nuclear genes, the expression of cell proliferation-related genes, and cell growth in endothelial cells (Figure 1E–H). These data suggest that IGFBP7 plays a crucial role in endothelial cell proliferation. Aplnr is mainly expressed on the surface of endothelial cells [24], and our previous study also found that IGFBP7 is primarily differentially expressed in Aplnr+ endothelial cells in the ALI model, but not in other subtypes of endothelial cells [16]. To further clarify the role of IGFBP7 in pulmonary blood vessels, using AAV-shIGFBP7 virus and Aplnr-Dre mice, we constructed endothelial IGFBP7 conditional knockdown mice and investigated the role of IGFBP7 in endothelial proliferation (see the Method section for detailed steps, Supplementary Figure S1A). The effect of IGFBP7 knockdown on endothelial cells in the ALI model was confirmed (Supplementary Figure S1B–E). We first evaluated the effect of IGFBP7 knockdown on endothelial cells in ALI model. We found that the knockdown of IGFBP7 resulted in a significant down-regulation of partial endothelial proliferation-associated genes (*Ccnc*, *Ccne1*, *Sox17*, *Egfr*, and *Ki67*, Figure 2A) and suppression of endothelial cell proliferation (Figure 2B,C) in the repair phase of ALI (7-day post-injury, 7dpi). This phenomenon was also partially observed in the acute phase of ALI (*Ccnd1*, *Ccne1*, *Egfr*, *Ki67*, and 1dpi, Figure 2A–C). The above results suggest that the knockdown of IGFBP7 is detrimental to the proliferative function of cells.

We then examined the effect of endothelial IGFBP7 knockdown on cell proliferation in healthy mice. We found that in healthy mice, the knockdown of IGFBP7 down-regulated the expression of some endothelial proliferation-associated genes (*Ccnd1*, *Ccne1*, *Sox17*, and *Ki67*, Figure 2D), even though there was no significant decrease in pulmonary vascular endothelial proliferation in mice (NC vs. AAV in 1 dpi: *P*=0.1965, NC vs. AAV in 7 dpi: *P*=0.1049, Figure 2E,F). The relatively insignificant effect of IGFBP7 knockdown on proliferation in healthy mice may be caused by the fact that IGFBP7 mainly regulates glycolipid metabolism in healthy organisms [25,26]. Taken together, the above results suggest that IGFBP7 knockdown inhibits the proliferation of pulmonary vascular endothelial cells in ALI, especially during the recovery period of ALI. However, we also observed that supplementation of rhIGFBP7 did not promote endothelial cell proliferation in both the ALI model and the healthy control group (Supplementary Figure S2A–D). This could be due to the direct supplementation of IGFBP7 leading to damage to the endothelial barrier (Supplementary Figure S2E and S2F) [16,27,28].

### IGFBP7 significantly regulates the expression of YAP1

**Figure 3:**
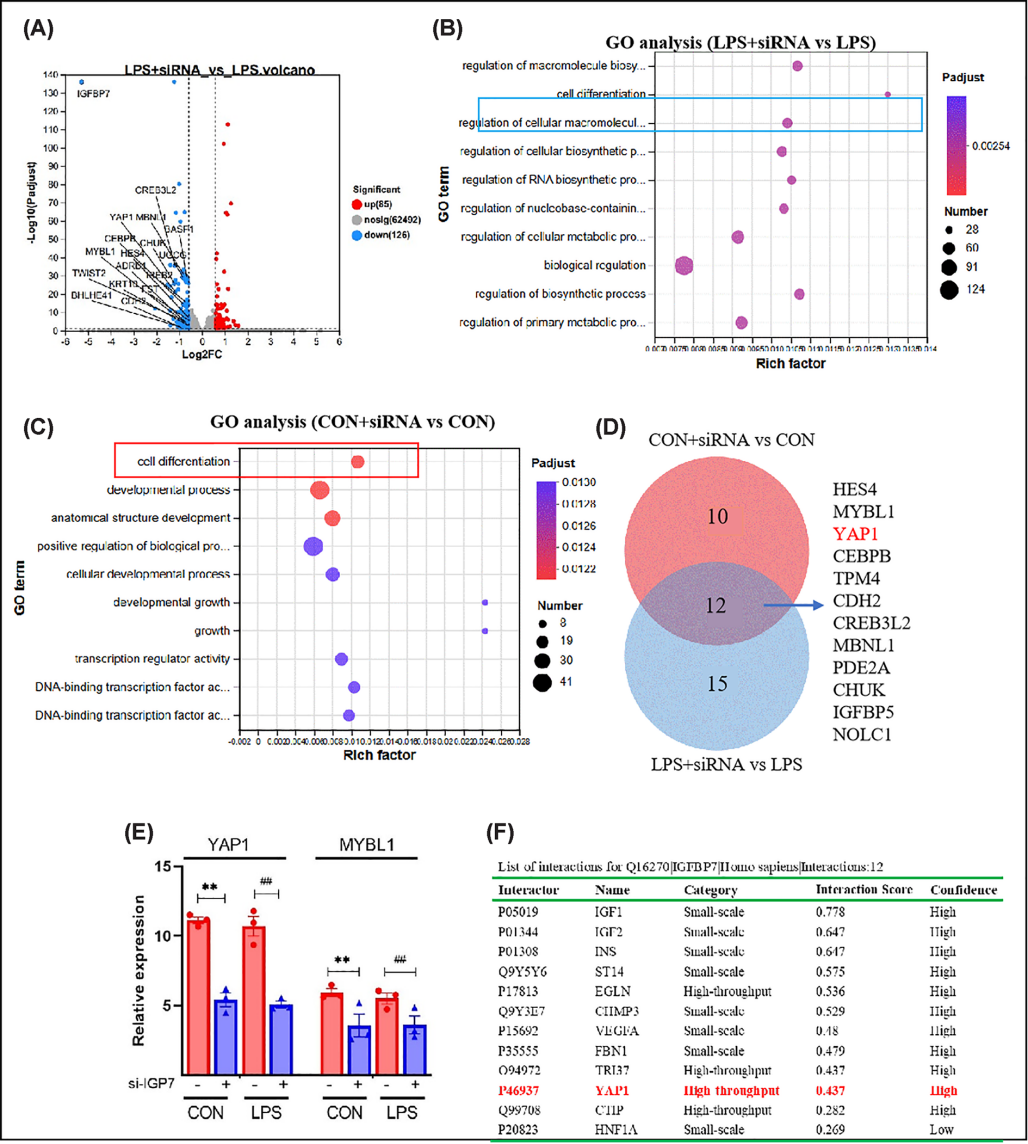
IGFBP7 regulates cell proliferation in association with YAP1 (**A**) Volcano map of differentially expressed genes between LPS+siRNA vs. LPS+NC groups. (**B**) Functional enrichment map of differentially expressed genes between LPS+siRNA vs. LPS+NC groups. (**C**) Functional enrichment map of differentially expressed genes between CON+siRNA vs. CON+NC groups. (**D**), The common gene in different proliferation-related clusters (27 in the LPS+siIGFBP7 vs. LPS group and 22 in the CON+siIGFBP7 vs. CON group). (**E**) Knockdown of IGFBP7 significantly reduced YAP1 mRNA levels: The TPM value was expressed as the mean ± S.E.M., *n*=3. (**F**) YAP1 was predicted as a potential interacting protein for IGFBP7 by the *HitPredict* database. CON, Control group; TPM, Transcript per Kilobase per Million mapped reads. ** represents the significance of Treat versus NC in the Control mouse model, and ## represents the significance of Treat versus NC in the LPS-treated mouse model. **P<0.01,##P<0.01.

To investigate the molecular mechanism by which IGFBP7 regulates endothelial cell proliferation, we did bulk RNA sequencing after the knockdown of IGFBP7 in endothelial cells. We first analyzed the effect of IGFBP7 knockdown in cell injury model. We identified 211 differentially expressed genes (DEGs), of which 85 were up-regulated and 126 were down-regulated. Among the DEGs, the knockdown of IGFBP7 significantly down-regulated genes such as *YAP1* [29], *MYBL1* [30], *CREB* [31], and *MBNL1* [32] (Figure 3A), which have been reported to be mainly involved in cell proliferation and differentiation. Functional enrichment analysis of the DEGs also revealed that the differential genes were significantly enriched in signaling pathways such as endothelial cell proliferation and cell growth (Figure 3B). We also analyzed the effect of IGFBP7 knockdown in healthy endothelial cells, and the results were similar to those above, with the differentially expressed genes mainly enriched in signaling pathways such as cell differentiation, growth, and development (Figure 3C). We then further analyzed the cell proliferation-related clusters (27 in the LPS+siIGFBP7 vs. LPS group and 22 in the CON+siIGFBP7 vs. CON group). We found that 12 genes were identified in both clusters (Figure 3D), and the YAP1 gene was the most differentially expressed (Figure 3E). In addition, we predicted similar results in the *HitPredict* database (Figure 3F), further suggesting the important role played by YAP1 in IGFBP7 function.

### IGFBP7 regulates the expression of YAP1-related molecules

**Figure 4:**
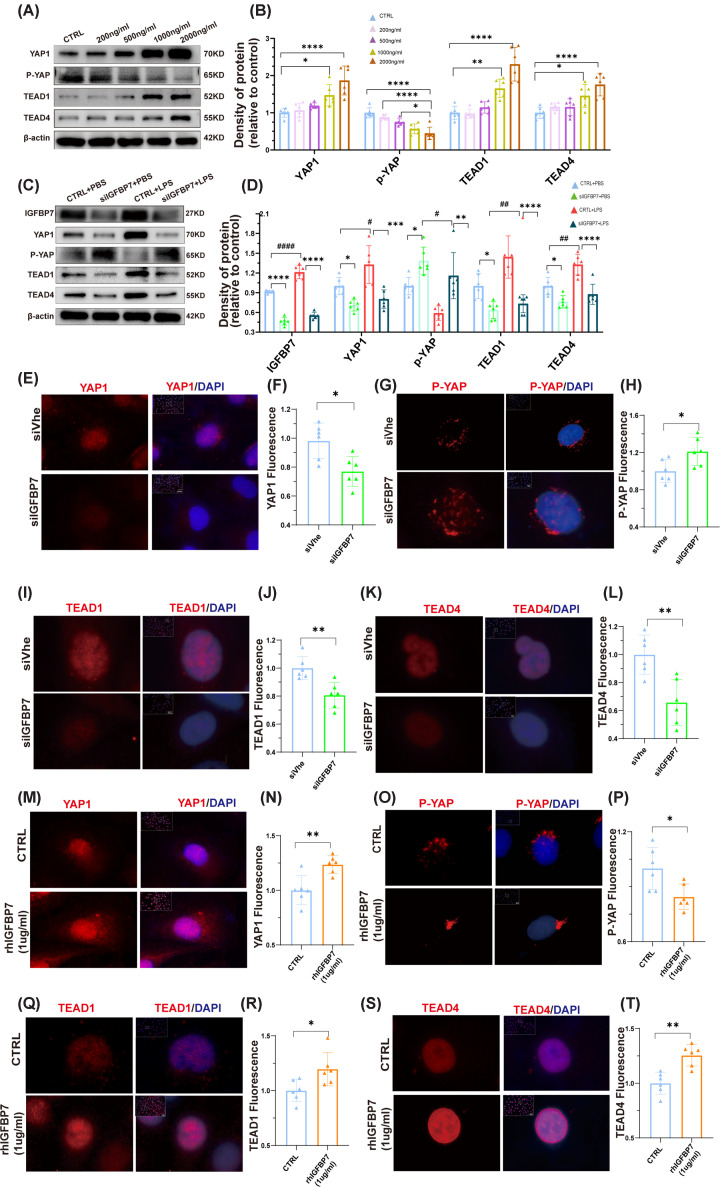
Activation of the YAP/TEAD1/TEAD4 signaling pathway in HUVECs by IGFBP7 (**A,B**) Detected by Western blotting, the protein expression of YAP1, P-YAP, TEAD1, and TEAD4 in HUVECs intervened with rhIGFBP7 (0, 200, 500, 1000, and 2000 ng/ml) for 48 h, internal control for normalization: β-Actin. (**C,D**) Detected by Western blotting, the protein expression of IGFBP7, YAP1, P-YAP, TEAD1, and TEAD4 in HUVECs transfected with IGFBP7 siRNA for 48 h and treated with LPS (5 µg/ml) for 24 h, internal control for normalization: β-Actin. (**E–L**) Representative images of YAP, P-YAP, TEAD1, TEAD4-AF555, and DAPI co-staining in HUVECs transfected with IGFBP7 siRNA for 48 h. Scale bars: 50 μm (40×). (**M**–**T**) Representative images of YAP, P-YAP, TEAD1, TEAD4-AF555, and DAPI co-staining in HUVECs intervened with rhIGFBP7 (1 μg/ml) for 48 h. Scale bars: 50 μm (40×). Data represented as means ± SDs. *P<0.05, **P<0.01, ***P<0.005,****P<0.001 [one-way ANOVA, Tukey’s test (B,D) and *t*-test (F,H,J,L,N,P,R,T)]. CTRL, Control group. * represents the significance of Treat versus NC in the Control mouse model, and # represents the significance of Treat versus NC in the LPS-treated mouse model. #P<0.05".Please add " ##P<0.01, ####P<0.001.

To further validate the relationship between YAP1 and IGFBP7, we first analyzed the regulatory effects of different concentrations of rhIGFBP7 intervention on YAP1-related proteins. We found that rhIGFBP7 significantly up-regulated YAP1 expression and increased rhIGFBP7-promoted YAP1 expression in a concentration-dependent manner (Figure 4A,B). Phosphorylated YAP1 (p-YAP1) is retained in the cytoplasm or degraded, thus limiting YAP activity. In contrast, unphosphorylated YAP1 enters the nucleus and binds to the transcription factors TEADs, which regulate the transcription of a variety of genes, thereby promoting tissue growth [18]. There are various isoforms of transcription factor TEADs, among which TEAD1 and TEAD4 are most closely related to lung injury [33,34]. We then detected the expression of p-YAP1 and found that p-YAP1 showed a concentration-dependent decrease (Figure 4A,B). In addition, increased rhIGFBP7 also promoted the expression of TEAD1 and TEAD4 in a concentration-dependent manner (Figure 4A,B). We then analyzed the expression of YAP1-related molecules in endothelial injury models. Consistent with previous research [27], our research also found that LPS stimulation increases the expression of IGFBP7 in endothelial cells [16]. Furthermore, LPS stimulation promotes the expression of YAP1, TEAD1, and TEAD4 molecules while reducing the expression of intracellular p-YAP1 in endothelial cells (Figure 4C,D). This indicates that inflammatory stimulation activates the IGFBP7 and YAP-TEAD signaling axis. We then found that the knockdown of IGFBP7 significantly decreased the expression of YAP1, TEAD1, and TEAD4 molecules, suggesting that the knockdown of IGFBP7 inhibits the endothelial YAP-TEAD axis in the injured endothelial model (Figure 4C,D). Similar results were also observed in the healthy control model (Figure 4C,D). Considering the functional differences of YAP1, TEAD1, and TEAD4 molecules at different cellular locations, we further localized YAP1, TEAD1, and TEAD4 molecules using immunofluorescence. The results revealed that knockdown of IGFBP7 reduced the accumulation of YAP1 (Figure 4E,F), TEAD1 (Figure 4I,J), and TEAD4 (Figure 4K,L) in the nucleus, while rhIGFBP7 increased the levels of intranuclear YAP1 (Figure 4M,N), TEAD1 (Figure 4Q,R) and TEAD4 (Figure 4S,T). These results suggest that IGFBP7 regulates the activation of the YAP1-TEAD pathway.

**Figure 5:**
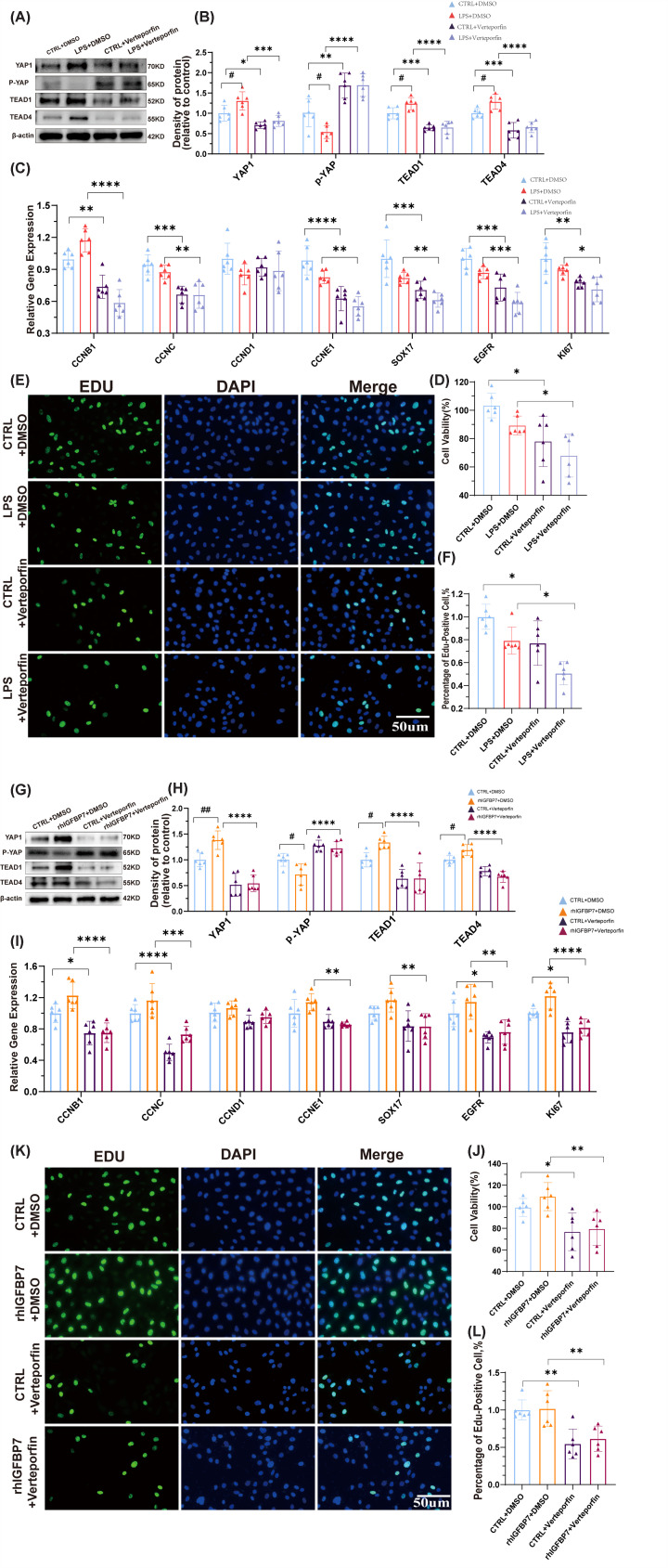
YAP1 inhibitors suppress cell proliferation by alleviating the activation of the YAP/TEAD1/TEAD4 signaling pathway mediated by IGFBP7 in HUVECs (**A,B**) Detected by Western blotting, the protein expression of YAP1, P-YAP, TEAD1, and TEAD4 in HUVECs treated with Verteporfin (1 μM, a YAP1 inhibitor) for 48 h, simultaneously with a 24 h LPS treatment, internal control for normalization: β-Actin. (**C**) Assessed by qRT-PCR, the mRNA expression of CCNB1, CCNC, CCND1, CCNE1, SOX17, EGFR, and KI67 in HUVECs intervened with Verteporfin (1 μM) for 48 h, concurrently treated with LPS (5 μg/ml) for 24 h. (**D**) Determined by the CCK-8 assay, the cell viability of HUVECs intervened with Verteporfin (1 μM) for 48 h, concurrently treated with LPS (5 μg/ml) for 24 h. (**E,F**) Representative images of EDU-AF488 and DAPI co-staining in HUVECs intervened with Verteporfin (1 μM) for 48 h, concurrently treated with LPS (5 μg/ml) for 24 h. Scale bars: 50 μm (40×). (**G,H**) Detected by Western blotting, the protein expression of YAP1, P-YAP, TEAD1, and TEAD4 in HUVECs treated with rhIGFBP7 (1 μg/ml) for 48 h, simultaneously intervened with Verteporfin (1 μM) for 48 h, internal control for normalization: β-Actin. (**I**) Assessed by qRT-PCR, the mRNA expression of CCNB1, CCNC, CCND1, CCNE1, SOX17, EGFR, and KI67 in HUVECs treated with rhIGFBP7(1 μg/ml) for 48 h, simultaneously intervened with Verteporfin (1 μM) for 48 h. (**J**) Determined by the CCK-8 assay, the cell viability of HUVECs treated with rhIGFBP7 (1 μg/ml) for 48 h simultaneously intervened with Verteporfin (1 μM) for 48 h. (**K,L**) Representative images of EDU-AF488 and DAPI co-staining in HUVECs treated with rhIGFBP7 for 48 h, simultaneously intervened with Verteporfin (1 μM) for 48 h. Scale bars: 50 μm (40×). Data represented as means ± SDs. *P<0.05, **P<0.01, ***P<0.005,****P<0.001 [one-way ANOVA, Tukey’s test (B,C,D,F,H,I,J,L)]. CTRL, Control group. * represents the significance of Treat versus NC in the Control mouse model, and # represents the significance of Treat versus NC in the LPS-treated mouse model. #P<0.05".Please add " ##P<0.01.

To further investigate the network relationship between IGFBP7 and YAP1, we first treated cells with Verteporfin, a YAP inhibitor, and examined the expression of YAP-related molecules. First, we found that Verteporfin significantly inhibited the expression of LPS-activated YAP1, TEAD1, and TEAD4 but up-regulated the expression of p-YAP1 (Figure 5A,B) in the injured endothelial model. Moreover, Verteporfin mildly down-regulated the expression of YAP, TEAD1, and TEAD4 molecules while up-regulating the expression of p-YAP1 (Figure 5A,B) in the healthy control model. Verteporfin also inhibited the expression of some proliferation-related genes (Figure 5C), cell proliferation (Figure 5D), and activation of nuclear genes (Figure 5E,F) in both injury models and healthy control models. This suggests that IGFBP7 may regulate cell proliferation through the YAP-TEAD signaling axis in both injury models and healthy control models. To further validate the interplay between IGFBP7, YAP1, and endothelial cell proliferation, we initially treated cells with the YAP inhibitor Verteporfin and then induced the up-regulation of YAP1, TEAD1, and TEAD4 molecules using rhIGFBP7. We found that Verteporfin blocked the rhIGFBP7-induced up-regulation of YAP1, TEAD1, and TEAD4 molecules while promoting the expression of p-YAP1 (Figure 5G,H). In addition, the expression of endothelial cell proliferation-related genes was significantly reduced (Figure 5I), and cell proliferation (Figure 5J) and activation of nuclear genes (Figure 5K,L) were also significantly inhibited in the combination of Verteporfin and rhIGFBP7 treatment group compared with the rhIGFBP7 intervention group. These results indicate that IGFBP7 regulates cell proliferation through a pathway mediated by YAP1.

### IGFBP7 promotes YAP1 up-regulation and cell proliferation via c-FOS molecule

**Figure 6:**
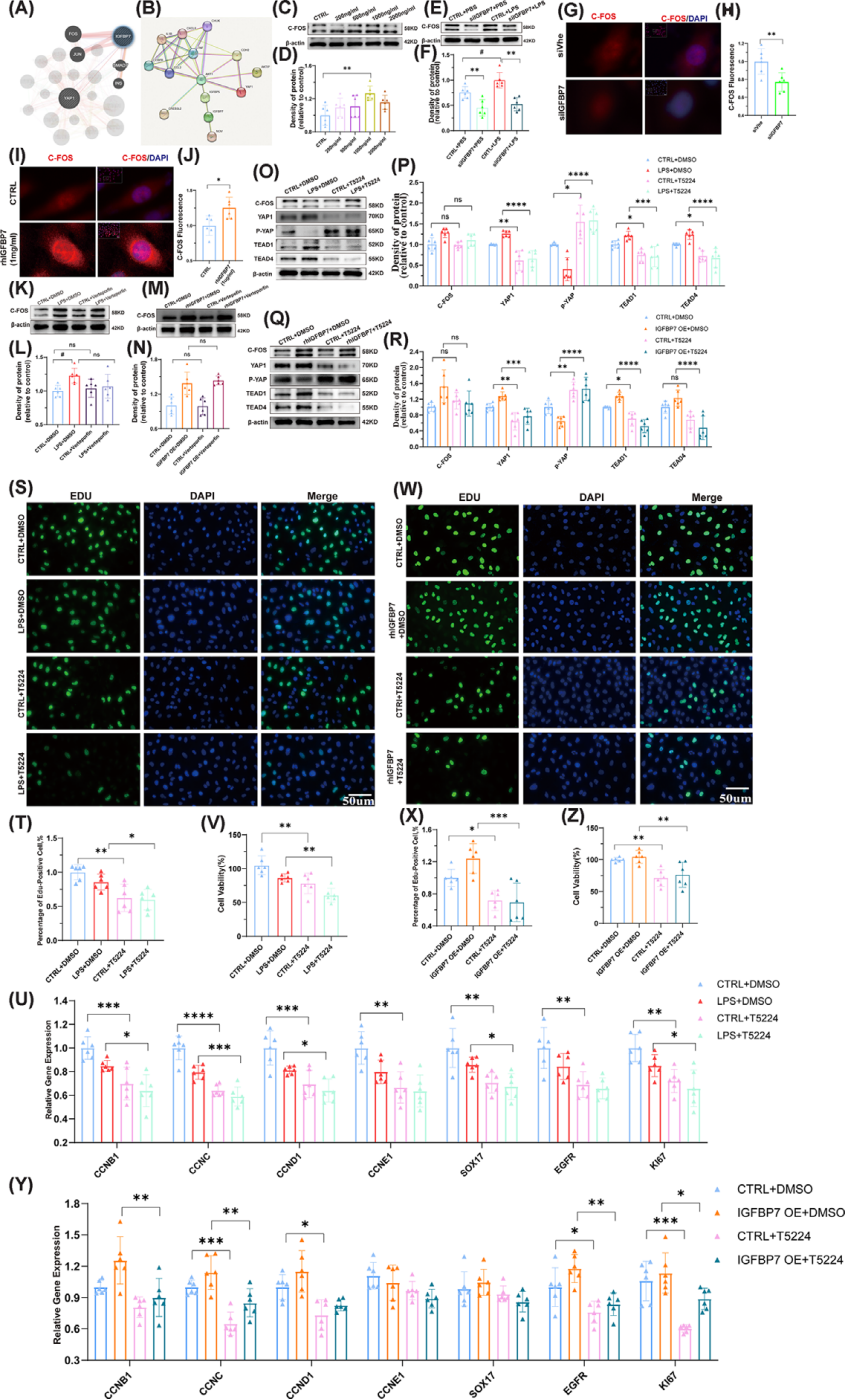
The C-FOS inhibitors suppress cell proliferation by relieving the IGFBP7-mediated activation of the C-FOS/YAP/TEAD 1-TEAD 4 signaling pathway in the HUVECs (**A,B**) *String* and *Genemania* databases were used to predict the potential regulatory molecules of IGFBP7. (**C,D**) Detected by Western blotting, the protein expression of c-FOS in HUVECs intervened with rhIGFBP7 (0, 200, 500, 1000, and 2000 ng/ml) for 48 h, internal control for normalization: β-Actin. (**E,F**) Detected by Western blotting, the protein expression of c-FOS in HUVECs transfected with IGFBP7 siRNA for 48 h and treated with LPS (5 µg/ml) for 24 h, internal control for normalization: β-Actin. (**G,H**) Representative images of c-FOS-AF555 and DAPI co-staining in HUVECs transfected with IGFBP7 siRNA for 48 h. Scale bars: 50 μm (40×). (**I,J**) Representative images of c-FOS-AF555 and DAPI co-staining in HUVECs intervened with rhIGFBP7 (1 μg/ml) for 48 h. Scale bars: 50 μm (40×). (**K–L**) Detected by Western blotting, the protein expression of c-FOS in HUVECs treated with Verteporfin (1 μM, a YAP1 inhibitor) for 48 h, simultaneously with a 24 h LPS treatment, internal control for normalization: β-Actin. (**M,N**) Detected by Western blotting, the protein expression of c-FOS in HUVECs treated with rhIGFBP7 (1 μg/ml) for 48 h, simultaneously intervened with Verteporfin (1 μM) for 48 h, internal control for normalization: β-Actin. (**O,P**) Detected by Western blotting, the protein expression of C-FOS, YAP1, P-YAP, TEAD1, and TEAD4 in HUVECs treated with T5224 (40 μM, a C-FOS inhibitor) for 48 h, simultaneously with a 24 h LPS treatment, internal control for normalization: β-Actin. (**Q,R**) Detected by Western blotting, the protein expression of C-FOS, YAP1, P-YAP, TEAD1, and TEAD4 in HUVECs treated with rhIGFBP7 (1 μg/ml) for 48 h, simultaneously intervened with T5224 (40 μM, a C-FOS inhibitor) for 48 h, internal control for normalization: β-Actin. (**S,T**) Representative images of EDU-AF488 and DAPI co-staining in HUVECs intervened with T5224 (40 μM) for 48 h, concurrently treated with LPS (5 μg/ml) for 24 h. Scale bars: 50 μm (20×). (**U**) Assessed by qRT-PCR, the mRNA expression of CCNB1, CCNC, CCND1, CCNE1, SOX17, EGFR, and KI67 in HUVECs intervened with T5224 (40 μM) for 48 h, concurrently treated with LPS (5 μg/ml) for 24 h. (**V**) Determined by the CCK-8 assay, the cell viability of HUVECs intervened with T5224 (40 μM) for 48 h, concurrently treated with LPS (5 μg/ml) for 24 h. (**W,X**) Representative images of EDU-AF488 and DAPI co-staining in HUVECs treated with rhIGFBP7 (1 μg/ml) for 48 h and simultaneously intervened with T5224 (40 μM) for 48 h. Scale bars: 50 μm (40×). (**Y**) Assessed by qRT-PCR, the mRNA expression of CCNB1, CCNC, CCND1, CCNE1, SOX17, EGFR, and KI67 in HUVECs treated with rhIGFBP7 (1 μg/ml) for 48 h and simultaneously intervened with T5224 (40 μM) for 48 h. (**Z**) Determined by the CCK-8 assay, the cell viability of HUVECs treated with rhIGFBP7 (1 μg/ml) for 48 h and simultaneously intervened with T5224 (40 μM) for 48 h. Data represented as means ± SDs. *P<0.05, **P<0.01, ***P<0.005,****P<0.001 [one-way ANOVA, Tukey’s test (D,F,L,N,P,R,T,U,V,X,Y,Z)] and *t*-test (H,J). CTRL, Control group.

Activator protein-1 (AP-1) mainly consists of c-Fos and c-Jun heterodimers, which are closely related to cell proliferation. AP-1 is expressed in normal tissues but is highly expressed in cells such as tumors [35]. Next, to investigate the molecular mechanism of IGFBP7 up-regulation of YAP1, we used *String* and *Genemania* databases to predict the possible regulatory molecules of IGFBP7. We found that c-FOS, c-JUN, and YAP1 molecules were retrieved in both databases, suggesting that IGFBP7 may interact closely with those molecules (Figure 6A,B). Next, we focused on the transcription factors c-Fos and c-Jun. We examined the regulatory effect of IGFBP7 on c-Fos, and the results showed that rhIGFBP7 partially up-regulated the level of c-Fos in a concentration-dependent manner (Figure 6C,D), while knockdown of IGFBP7 decreased the expression of c-Fos in both injured endothelial model and healthy endothelium (Figure 6E,F). We also performed immunofluorescence analysis to localize c-Fos. The results showed that the knockdown of IGFBP7 primarily reduced the expression of nuclear c-Fos (Figures 6G,H). Moreover, the nuclear c-Fos expression was increased after rhIGFBP7 intervention in endothelial cells (Figure 6I,J). We then analyzed the role of c-Fos in regulating YAP1 by IGFBP7. The results showed that verteporfin did not significantly affect the c-Fos level (Figure 6K–N), suggesting that c-Fos may be the upstream effector of YAP1. T-5224 is a selective inhibitor of the transcription factor c-Fos, which specifically inhibits the DNA-binding activity of c-Fos/c-Jun without affecting the binding activity of other transcription factors. To further validate the relationship between c-Fos and YAP1, we tested the regulation of YAP-related genes by administration of T-5224. We found that T-5224 suppressed the level of YAP1 and the expression of TEAD1 and TEAD4 (Figure 6O–R). T-5224 partially reversed the up-regulation of YAP, TEAD1, and TEAD4 caused by LPS stimulation or rhIGFBP7 treatment (Figure 6O–R). We also evaluated the effect of T-5224 on endothelial cell proliferation. The results showed that T-5224 inhibited the activation of nuclear genes (Figure 6S,T), expression of proliferation-related genes (Figure 6U), and cell proliferation (Figure 6V) in both the injured endothelial model and the healthy control model. Furthermore, T-5224 partially alleviated the activation of nuclear genes (Figure 6W,X), expression of proliferation-related genes (Figure 6Y), and cell proliferation (Figure 6Z) induced by rhIGFBP7 treatment. These data indicate that IGFBP7 partially promotes endothelial cell proliferation through the c-FOS-mediated pathway.

### IGFBP7 interacts with c-Fos and promotes c-Fos phosphorylation

**Figure 7:**
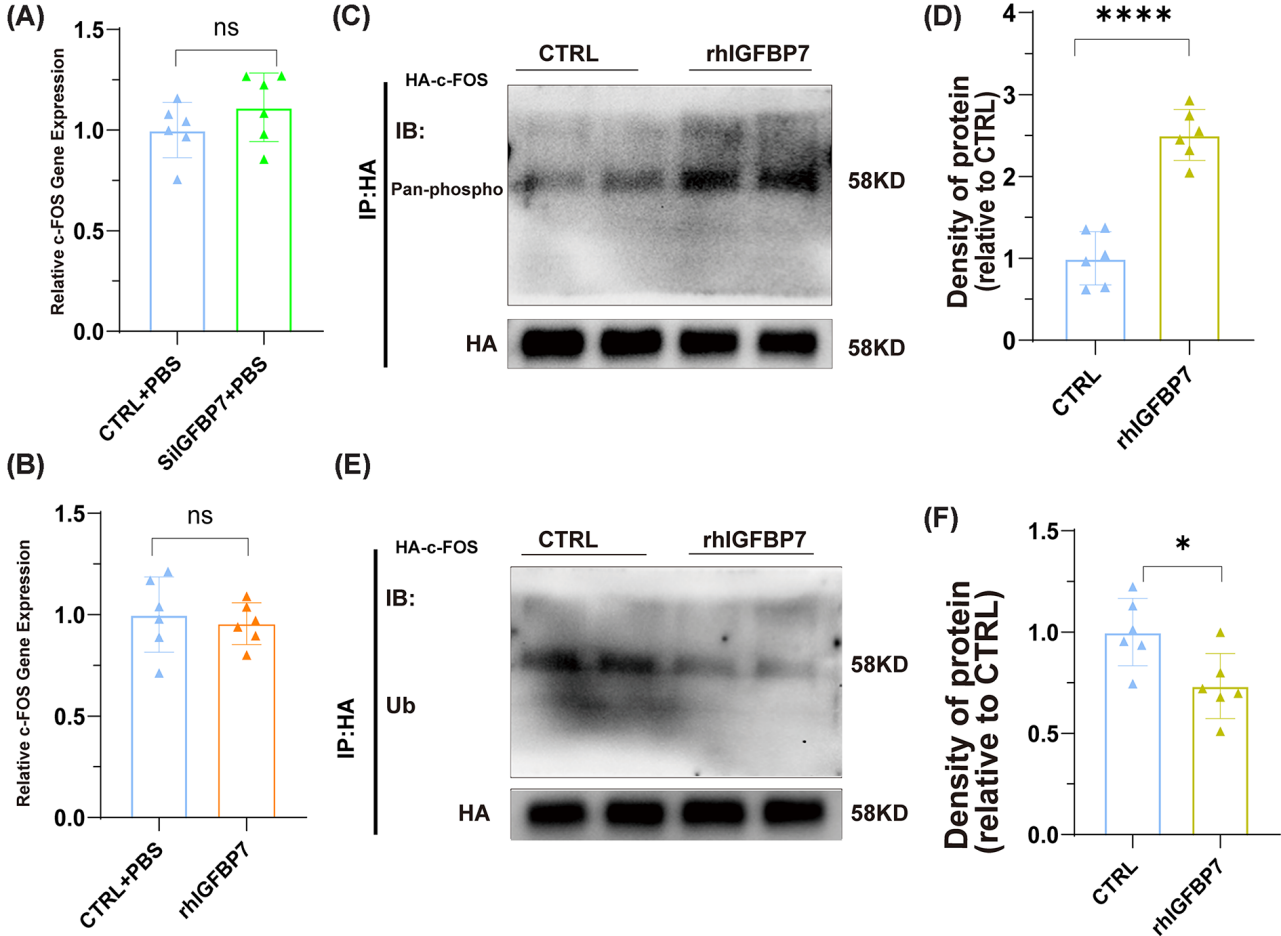
Up-regulation of C-FOS expression by IGFBP7 inhibiting ubiquitination and promoting phosphorylation of C-FOS (**A**) Assessed by qRT-PCR, the mRNA expression of c-FOS in HUVECs transfected with IGFBP7 siRNA for 48 h. (**B**) Assessed by qRT-PCR, the mRNA expression of c-FOS in HUVECs intervened with rhIGFBP7 (1 μg/ml). (**C–F**) Detected by Western blotting, the protein expression of Ubiquitin, pan Phospho-Serine in HUVECs transfected with the HA-Tag-C-FOS plasmid for 48 h, simultaneously intervened with rhIGFBP7 (1 μg/ml) for an additional 48 h, internal control for normalization: HA. Extraction of C-FOS protein using Anti-HA Magnetic Beads. Data represented as means ± SDs. *P<0.05, ****P<0.001 [*t*-test (A,B,D,F)]. CTRL, Control group.

To further explore the mechanism of c-Fos up-regulation by IGFBP7, we first examined the changes of c-Fos at the transcriptome level and found that IGFBP7 did not affect the mRNA level of c-Fos (Figure 7A,B). Post-translational modifications play an important role in regulating protein levels, and phosphorylated c-Fos is transcriptionally active and protected from degradation, so we investigated the role of IGFBP7 on c-Fos phosphorylation. We precipitated c-Fos protein and examined its phosphorylation level using immunoprecipitation assay and found that IGFBP7 significantly increased the phosphorylation level of c-Fos (Figure 7C,D), suggesting that IGFBP7 promotes c-Fos phosphorylation. Similarly, we also detected the ubiquitination level of c-Fos, and the results showed that IGFBP7 decreased the ubiquitination level of c-Fos (Figure 7E,F), suggesting that IGFBP7 inhibited the degradation of c-Fos. The above results indicate that IGFBP7 promotes phosphorylation and inhibits the degradation of c-Fos, resulting in increased c-Fos levels (Supplementary Figure S3).

### The down-regulation of IGFBP7 inhibits the activation of the C-FOS/YAP/TEAD1-TEAD4 signaling pathway *in vivo*

**Figure 8:**
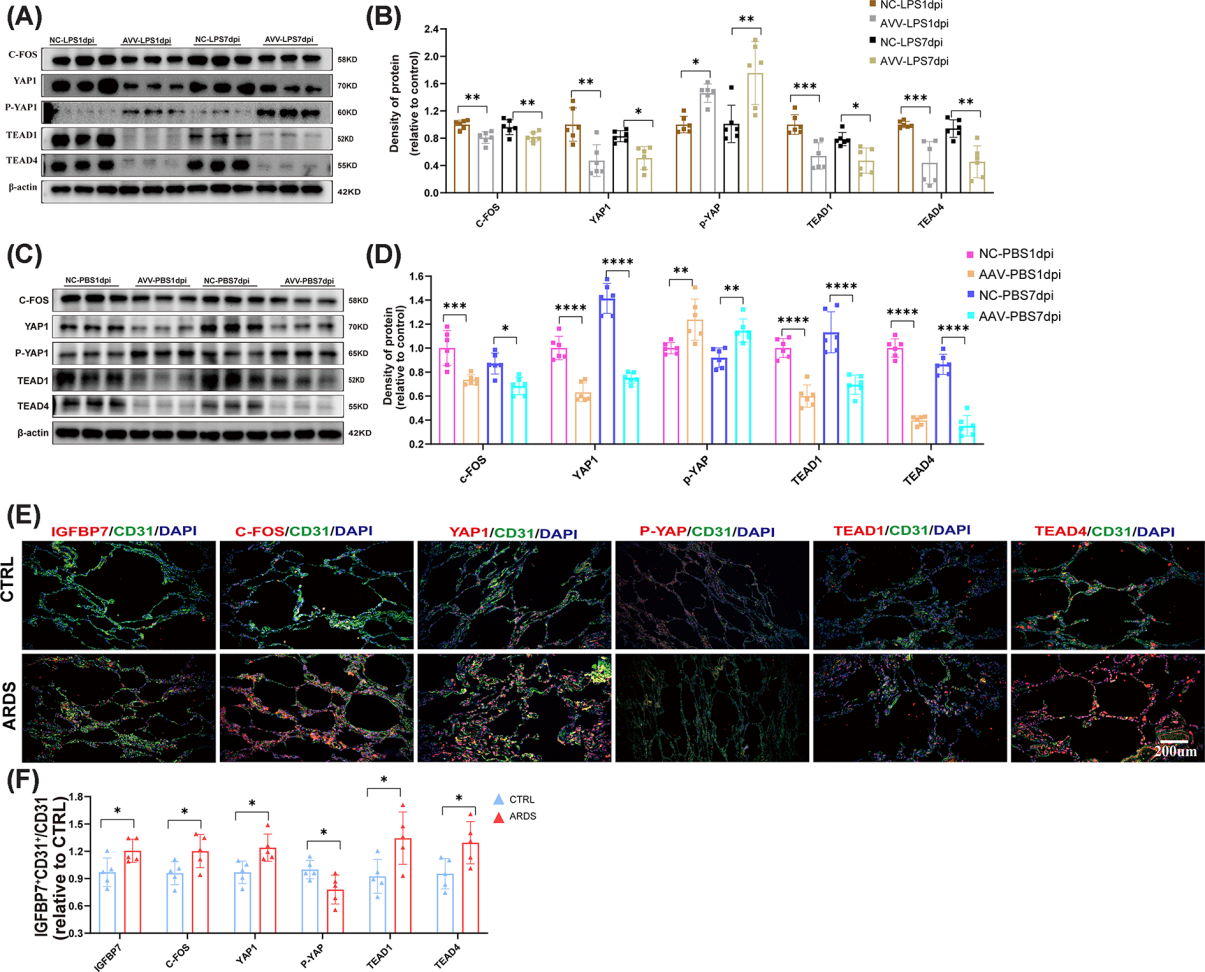
Down-regulation of IGFBP7 inhibits the activation of the C-FOS/YAP/TEAD1-TEAD4 signaling pathway *in vivo* (**A,B**) Detected by Western blotting, the protein expression of C-FOS, YAP1, P-YAP, TEAD1, and TEAD4 in mouse lung tissues from ordinary and IGFBP7-KO mice (obtained by endothelial cell-specific knockdown of IGFBP7 in Aplnr-2A-DreERt2 mice) subjected to intratracheal injection with saline treatment (Collecting lung tissues after 24 h or 7 days), internal control for normalization: β-Actin. (**C,D**) Detected by Western blotting, the protein expression of C-FOS, YAP, P-YAP, TEAD1, and TEAD4 in mouse lung tissues from ordinary and IGFBP7-KO mice subjected to intratracheal injection with LPS (5 mg/kg) treatment (Collecting lung tissues after 24 hours or 7 days), internal control for normalization: β-Actin. (**E,F**) Representative images of IGFBP7, C-FOS, YAP, P-YAP, TEAD1, TEAD4-AF555, CD31-AF488, and DAPI co-staining in ARDS human lung tissue. Data represented as means ± SDs. *P<0.05, **P<0.01, ***P<0.005,****P<0.001 [one-way ANOVA, Tukey’s test (**B,D**)] and t-test (**F**). CTRL, Control group.

To verify whether the effect of IGFBP7 on the c-Fos/YAP/TEAD1-TEAD4 signaling axis *in vivo* is consistent with that observed *in vitro*, we used conditional IGFBP7 knockdown mice in endothelial cells to study the effects of IGFBP7 knockdown on the expression of c-Fos, YAP, TEAD1, and TEAD4 proteins *in vivo*. We found that IGFBP7 knockdown inhibited the activation of the c-Fos/YAP/TEAD1/TEAD4 signaling axis in both the acute phases (1 dpi) and recovery phases (7 dpi) of lung injury (Figure 8A,B). In the healthy control model, decreased expression of IGFBP7 also led to reduced expression of c-Fos, YAP, TEAD1, and TEAD4 proteins (Figure 8C,D). Previous studies have shown that ARDS patients have elevated levels of IGFBP7 in their plasma [36]. To further validate the role of IGFBP7 on the c-Fos/YAP/TEAD1/TEAD4 signaling axis in human lungs, we analyzed the expression of IGFBP7/c-Fos/YAP/TEAD1/TEAD4 in lung tissues of ARDS patients and para-cancerous tissues of lung cancer patients collected previously [16]. We detected c-Fos/YAP/TEAD1/TEAD4 expression. We found that IGFBP7, c-Fos, YAP1, TEAD1, and TEAD4 expression was higher in the lung tissues of ARDS patients than in control patients (Figure 8E,F). These results suggest that consistent with* in vitro* experiments, IGFBP7 activates the c-Fos/YAP/TEAD1/TEAD4 signaling axis in both mouse models and ARDS lung tissues.

## Discussion

IGFBP7 is emerging as a potential therapeutic target for tumors and as one of the detrimental factors that exacerbate acute inflammatory injury, such as acute kidney injury and acute lung injury. Our previous results suggest that IGFBP7 aggravates inflammation-induced endothelial damage in the acute phase. However, the role of IGFBP7 in the recovery phase of ALI remains unclear. In the present study, we found that IGFBP7 promotes endothelial cell proliferation and vascular repair during the recovery phase of ALI. Mechanistically, IGFBP7 promotes transcriptional activation of proliferation-related genes through c-Fos-mediated activation of YAP-TEAD signaling. The inhibition of c-Fos or YAP also blocked the pro-proliferative effects of IGFBP7. Our study found that IGFBP7 promotes endothelial cell repair during the recovery phase of ALI, which broadens our understanding of the role of IGFBP7 in different stages of ALI and suggests that IGFBP7 as a potential therapeutic target in ALI needs to take into account the period specificity of ALI.

IGFBP7 has been found to play diverse and vital roles in different cells. Studies have reported that IGFBP7 promotes the proliferation, differentiation, and migration of glioma cells [9], keratinocyte [37], and bone marrow mesenchymal stem cells [10,38] and participates in exercise-triggered myocyte protection, thereby improving myocyte function [39]. IGFBP7 also promotes tumor vascularization and formation, favoring tumor metastasis, while anti-IGFBP7 inhibits tumor vascularization and angiogenesis [11]. In addition, IGFBP7 also inhibits cell proliferation. For example, Zhang et al. [40] found that IGFBP7 inhibits the proliferation and cell cycle of thyroid cancer cells. A recent study has reported that IGFBP7 exacerbates the pathological processes in the acute phase of inflammatory diseases, such as acute kidney injury and acute lung injury, and is one of the injury markers in acute kidney injury. However, the role of IGFBP7 in the recovery phase of inflammatory diseases is poorly understood. In ALI, pulmonary tissue injury increases IGFBP7 secretion and exacerbates sepsis-induced ALI by activating the ERK1/2 pathway [27]. Our previous studies have also shown that IGFBP7 exacerbates endothelial cell injury induced by acute-phase inflammation. In the present study, after the knockdown of IGFBP7 during the acute phase of ALI, we observed a reduction in endothelial cell proliferation-related genes but no significant inhibitory effect on endothelial cell proliferation. This may be attributed to the substantial impact of the inflammatory storm in the acute phase, in which immune cells such as macrophages and neutrophils play a significant regulatory role in the interaction with pulmonary vascular endothelial cells. In contrast, during the recovery phase of ALI, IGFBP7 significantly enhanced the expression of genes related to endothelial cell proliferation, promoting endothelial cell proliferation and repairing the vascular barrier. This may be due to the interaction between the reduction of acute inflammation during the recovery period and the activation of repair mechanisms after injury.

Multiple studies have reported that IGFBP7 is closely associated with human insulin resistance, diabetes, lipid metabolism, and obesity [25,41–43]. For example, recent studies have shown that IGFBP7 influences lipid accumulation and triglyceride production in mature adipocytes [44]. IGFBP7 binds to insulin receptors, thereby inducing lipogenesis and gluconeogenesis [45,46]. In our study, the knockdown of IGFBP7 in the healthy control model resulted in only a partial decrease in the expression of proliferation-related genes. In addition, the knockdown of IGFBP7 had no significant effect on the proliferation of pulmonary vascular endothelial cells in mice, suggesting that the pro-proliferative effect of IGFBP7 is not predominant in the healthy model. This may be because, under physiological conditions, IGFBP7 primarily regulates the organism’s glycolipid metabolism.

YAP1 is a critical downstream regulatory target of the Hippo signaling pathway. Its role in promoting tumor cell proliferation and inducing cancer stem cell properties has been well established [47]. In normal mouse liver, YAP induces hepatomegaly and regeneration [48]. YAP also participates in the proliferation of non-tumor cells such as intestinal stem cells [49], Schwann cells [50], and cardiomyocytes [51,52] to facilitate tissue and organ repair. Mechanistically, by analyzing transcriptome sequencing data in the endothelial injury model, we observed that cell proliferation-related pathways were significantly enriched and further identified that the transcript levels of YAP1 molecules were significantly down-regulated. We also observed that IGFBP7 dose-dependently promoted the expression of YAP1, TEAD1, and TEAD4. Knockdown of IGFBP7 reversed the up-regulation of YAP1, TEAD1, and TEAD4 caused by inflammatory injury. Using immunofluorescence localization, it was also found that IGFBP7 decreased the level of phosphorylated YAP1 in the cytoplasm and increased the entry of non-phosphorylated YAP1 into the nucleus, as well as increasing the levels of downstream TEAD1 and TEAD4 molecules in the nucleus. The inhibition of YAP1 blocks the regulatory effects of IGFBP7 on cell proliferation-related genes and the regulation of downstream molecules TEAD1 and TEAD4.

The critical role of activator protein 1, a transcriptional regulator mainly composed of the Fos and Jun families [53], has been widely elaborated in cell proliferation, transit, and death processes. Recent studies have also identified Fos as an upstream regulatory transcription factor of YAP1 that promotes the reprogramming process in which YAP1 is involved [54]. In the present study, the antagonists of Fos blocked the regulatory effects of IGFBP7 on YAP1 and its downstream molecules and blocked the regulation of cell proliferation and proliferation-related genes by IGFBP7. The above results suggest that the Fos-YAP1 pathway mediates the function of IGFBP7 in regulating cell proliferation.

However, our study also observed the regulation of other proliferation-related molecules, such as Mybl1 and Cebpb, by IGFBP7, suggesting that IGFBP7 regulates cell proliferation by different signaling pathways. Whether these unknown pathways significantly promote pulmonary endothelial cell proliferation during the recovery phase of ALI remains to be determined. Our study clarified the role of IGFBP7 in regulating lung endothelial cell proliferation through the Fos-YAP1 pathway *in vitro* and *in vivo* models. We also detected elevated expression of IGFBP7 in inflamed human lung endothelial cells and observed its potential activation of the Fos-YAP1 pathway. However, the lack of a human model of IGFBP7 knockdown or overexpression temporarily prevented the validation of the role of IGFBP7 in human ARDS. Easier availability of organoid technology in the future may further address this limitation. In addition, it has been shown that the interaction between CD93 and IGFBP7 promotes the formation of abnormal tumor vascular networks, and the use of IGFBP7 monoclonal antibodies may promote the normalization of the tumor vascular system, thereby enhancing drug delivery and improving the efficacy of immunotherapy. This offers the potential for further exploration of anti-IGFBP7 antibodies in treating vascular barrier dysfunction in the acute phase of ALI.

In summary, our research findings suggest that IGFBP7 promotes endothelial cell repair during the recovery phase of ALI, most likely through activation of the YAP1 signaling pathway mediated by the Fos transcription factor. Combining the reported crucial role of IGFBP7 in the acute phase of ALI, our study reports the diverse roles of IGFBP7 in different stages of ALI, indicating the need to consider the stage-specificity of targeting IGFBP7 in the treatment of ALI. However, further molecular, safety, and clinical studies are still needed to validate these findings.

## Methods

**Table:** 

Reagent	Source	Identifier
IGFBP7 Rabbit mAb	Zenbio	Cat# R24669
C-Fos Rabbit pAb	Zenbio	Cat# 340249
YAP1 Rabbit mAb	Zenbio	Cat# R380482
Rb mAb to active YAP1	Abcam	Cat# Ab205270
Phospho-YAP (Ser127) Antibody	Affinity	Cat# AF3328
TEAD1 Rabbit mAb	Zenbio	Cat# R389264
TEAD4 Polyclonal antibody	Proteintech	Cat# 12418-1-AP
CD31/PECAM-1 Antibody (H-3)	Santa	Cat# sc-376764
Ubiquitin Rabbit pAb	Zenbio	Cat# 381080
pan Phospho-Serine/Threonine Polyclonal Antibody	Beyotime	Cat# AF5725
HA Tag Mouse Monoclonal Antibody	Beyotime	Cat# AF2858
Β-Actin	Santa	Cat# Sc-47778
Goat Anti-Mouse IgG H&L (Alexa Fluor® 488)	Abcam	Cat# ab150113
Goat Anti-Rabbit IgG H&L (Alexa Fluor® 555)	Abcam	Cat# ab150078
HRP-conjugated Goat Anti-Rabbit IgG(H+L)	Proteintech	Cat# SA00001-2
HRP-conjugated Goat Anti-mouse IgG(H+L)	Proteintech	Cat# SA00001-1
T-5224(C-FOS inhibitor)	MCE	Cat# HY-12270
Verteporfin (YAP1 inhibitor)	MCE	Cat# HY-B0146
Recombinant Human IGFBP-7 protein	Elabscience	Cat# PDMH100099
LPS (*Escherichia coli*, serotype O55:B5)	Sigma-Aldrich	Cat# L2880
SYBR Green qPCR Master Mix (No ROX)	MCE	Cat# HY-K0523
Tamoxifen	Beyotime	Cat# ST1682-10ML
Lipofectamine™ 3000	ThermoFisher	Cat# L3000001
Cell Counting Kit-8	MCE	Cat# HY-K0301
RNA total extraction kit	TIANGEN	Cat# Y1216
RT Master Mix for qPCR	MCE	Cat# HY-K0510
BeyoClick™ EdU-594Cell proliferation assay kit	Beyotime	Cat# C0078S
HA Label Protein Immunoprecipitation Kit	Beyotime	Cat# P2185S
**Experimental models: Cell lines**		
HUVECs	Meisen	Cat# CTCC-009-493
**Experimental models: Organisms/strains**		
C57BL/6J wild-type (WT) mice	Chongqing Medical University	N/A
Aplnr-2A-DreERT2 mice	SHANGHAI MODEL ORGANI SMS	N/A

### Human tissues

ARDS lung tissue and paracancer tissue in the lung were obtained from patients undergoing surgery at the Second Affiliated Hospital of Chongqing Medical University as approved by the Institutional Review Board (Approval No. Science Ethics Review 2024-001) and the consent of the patients or patient’s family with body organ donation. After obtaining the tissue, immediately immerse it in 4% paraformaldehyde for 24 h, followed by dehydration and embedding in paraffin for sectioning.

### Animals

All animal experiments were conducted in accordance with the guidelines from Directive 2010/63/EU of the European Parliament on the protection of animals used for scientific purposes. The experimental protocols were approved by the Ethics Committee for Animal Experiments and the Institutional Animal Care and Use Committee of the Second Affiliated Hospital of Chongqing Medical University (Approval No. IACUC-SAHCQMU-2023-0054). C57BL/6J wild-type (WT) mice were obtained from Chongqing Medical University. Aplnr-2A-DreERt2 mice were purchased from SHANGHAI MODEL ORGANI SMS. All animals were housed in a pathogen-free environment with the ambient temperature maintained at 21–23°C and relative humidity at 50–60%, with a 12:12 h light:dark cycle. Animals were allowed ad libitum access to water and food.

### ALI mouse models

We employed an ALI murine model of ALI induced by tracheal injection of 5 mg/kg LPS. Briefly, mice were injected with tamoxifen (75 mg/kg, intraperitoneally) to activate Dre recombinase in Aplnr-2A-DreERt2 mice, then continued to be fed for 4 weeks before AAV was injected via tail vein to induce endothelium-specific IGFBP7 knockdown. Mice were continued to be fed for 1 month and later used for subsequent experiments. Mice were anesthetized with pentobarbital sodium (50 mg/kg) and intratracheally administered phosphate-buffered saline (PBS) vehicle control or LPS (5 mg/kg, LPS from *Escherichia coli*). The mice were anesthetized 24 h or 7 d after LPS administration, and the lung tissues were collected. r30shRNA (IGFBP7) was provided by OBiO Technology (Shanghai) Corp., Ltd.

### Cell culture and treatment

The HUVECs cell line was obtained from Meisen Chinese Tissue Culture Collections (Zhejiang, China). The cells were cultured in Dulbecco’s Modified Eagle Medium (DMEM) (Gibco, China) containing 10% fetal bovine serum (Gibco, Australia) and 1% penicillin-streptomycin (Gibco, China) at 37°C in humidified incubator supplemented with 5% CO_2_. Cells were transfected with siRNA, plasmid, or IGFBP7 protein (1 μg/ml) and then stimulated with or without LPS (5 μg/ml). For signaling pathway inhibition experiments, HUVECs were pretreated with T-5224 (40 mM, C-FOS inhibitor) or Verteporfin (10 mM, YAP1 inhibitor).

### Cells transfection

In the transfected cells, cells at 50–70% confluence were transfected with siRNA or plasmid using OptiMEM and Lipofectamine 3000 (Invitrogen). Control siRNA and all siRNA targeting IGFBP7, HA-tag-C-FOS, and IGFBP7 were obtained from SangonBiotech (Shanghai, China).

### CCK-8 assay

Cell viability was measured using a CCK-8 kit according to the manufacturer’s instructions. HUVECs were seeded into 96-well plates (2 × 10^5^ cells/well) and pre-incubated at 37°C in a humidified atmosphere with 5% CO_2_ for 12 h. Following the indicated treatment, 10 μl CCK-8 mixed with 100 μl DMEM was added to each well and incubated for 2 h. The absorbance of each well was measured at 450 nm using a microplate reader (Thermo Scientific, Waltham, MA, U.S.A.). Cell viability was calculated using the following equation:

$$\text{cell viability}\;=\;\frac{\left({\text{OD}}_{\text{test}}\;-\;{\text{OD}}_{\text{blank}}\right)}{\left({\text{OD}}_{\text{control}}\;-\;{\text{OD}}_{\text{blank}}\right)}\times \;100\%$$

OD: Optical Density.

### Cell EDU

5-Ethynyl-2′-deoxyuridine (EDU), a thymine nucleoside analog, can replace thymine (T) in DNA replication during cell proliferation. Therefore, the detection of EDU+ cells by immunofluorescence indirectly reflects cell proliferation. Briefly, cells were seeded into 24-well plates at a 50–60% density (1 × 10^5^ cells/well) for the corresponding intervention and incubated with EDU (10 μM) for 2 h. Cells were fixed with 4% paraformaldehyde for 15 min, permeabilized using 0.3% triton-100 for 15 min, stained according to the EDU kit instructions, and stained nuclei with DAPI. Images were acquired using a fluorescence microscope and manually counted for EDU-positive cells and DAPI numbers.

### Immunofluorescence staining

The cells were fixed with 4% paraformaldehyde for 15 min, while the paraffin-embedded sections of both mouse and human tissues were deparaffinized and then subjected to heat-induced antigen retrieval using sodium citrate buffer. Subsequently, the sections were treated with Triton X-100 (0.3%) for 15 min to enhance membrane permeabilization. Next, the slide was blocked with fetal bovine serum (FBS) for 1 h. Then, the following primary antibodies were used to stain the slides and cells during overnight incubation at 4°C: anti-CD31(diluted 1:100), anti-C-FOS (diluted 1:100), anti-IGFBP7 (diluted 1:100), anti-YAP1 (diluted 1:100), anti-P-YAP1 (diluted 1:100), anti-TEAD1 (diluted 1:100), anti-TEAD4 (diluted 1:100), EDU. The next day, post-slides or cells were washed with PBS-Tween-100. The cells were then stained with anti-rabbit or anti-mouse fluorescent secondary antibody and incubated at room temperature for 2 h. After washing with PBS-Tween-100, nuclei were counter-stained with 4′,6′-diamidino-2-phenylindole (DAPI) was incubated for 10 min. Images were acquired using a fluorescence microscope and quantified on more than six sections using ImageJ software.

### Quantitative real-time PCR (RT-PCR)

Primers and sequences that need to be detected are shown in Supplementary Table S1. Total message RNA was extracted using TRIzol® Reagent (TIANGEN, DP451) according to the manufacturer’s protocol. Total RNA was quantified using the ND-2000 (NanoDrop, Thermo Scientific). cDNA was synthesized with a PrimeScript™ RT Reagent Kit with gDNA Eraser (MCE, USA). RT-PCR was performed using SYBR Green qPCR Master Mix (MCE, U.S.A.). The denaturation process is 95°C for 10 s, the annealing process is 60°C for 30 s, and the cycle is 35 times. The relative expression of mRNA was analyzed using the 2−ΔΔCt method.

### Western blotting analysis

Protein samples were detected using electrophoresis (BioRad) and immunoblotting. Briefly, proteins were extracted with RIPA with 1 mM PMSF (Beyotime, China), and concentrations of the supernatant were determined using a BCA protein assay kit (Beyotime, China). The concentration of polyacrylamide in this experiment was 10% or 12%, depending on the molecular weight size of the protein. The protein sample size was 10 μg. The proteins were separated by electrophoresis and transferred onto 0.45 μm polyvinylidene difluoride (PVDF) membranes. Next, the PVDF was blocked in 5% skimmed milk buffer for 1 h to minimize non-specific signals. After blocking, membranes were incubated with primary antibodies: anti-C-FOS (diluted 1:1000), anti-YAP1 (diluted 1:1000), anti-phospho-YAP (diluted 1:1000), anti-TEAD1 (diluted 1:1000), anti-TEAD4 (diluted 1:1000), anti-β-actin (diluted 1:1000), anti-Ubiquitin (diluted 1:1000), and anti-pan (diluted 1:1000). Blots were incubated with HRP-conjugated secondary antibody (diluted 1:5000) for 1 h and treated with ECL substrate (Beyotime, China). ImageJ was used for densitometric analysis of protein bands.

### Co-immunoprecipitation

The HUVECs cells were transfected with HA-Tag-C-FOS plasmid. The control group used PBS intervention, and the experimental group used IGFBP7 intervention after 48 h of incubation. Cells were lysed using 200 μl of cell lysate containing protease inhibitor and phosphatase inhibitor, added 20 μl Anti-HA Magnetic Beads, and incubated with a shaker 4°C overnight. It was then placed on a magnetic stand for 10 min, washed repeatedly three times with protein phosphatase inhibitor lysate, then added 5X SDS-PAGE Sample Loading Buffer (P0285-15ml, Beyotime), and heated for 5 min at 95°C, separated on a magnetic stand for 10 s, and the supernatant was removed for Western blot analysis.

### Online database analysis

The *String* and *Genemania* databases were used to find correlations between IGFBP7 and other genes. The *String* and *Genemania* databases are primarily used to explore gene interactions and predict gene functions. The website URLs are https://cn.string-db.org/ and https://genemania.org/, respectively.

### RNA-sequence

Major Biotechnology Co., Ltd. conducted RNA library preparation, high-throughput sequencing, and data analysis. The RNA total extraction kit (TIANGEN) was used to extract total RNA from HUVECs with IGFBP7 knockdown by siRNA and normal HUVECs. Total RNA was also collected from both groups after the LPS intervention. In the present study, 3 μg of RNA was used as the input material for each sample. The sequencing experiment employed the Illumina NovaSeq Reagent Kit method for library construction, with index codes added to attribute sequences to each sample. Sequencing was performed on the Illumina NovaSeq 6000 platform, generating paired-end reads of 150 bp. Differential gene expression analysis was conducted using the DEseq2 algorithm, filtering for genes with a fold change >2 and a *P*-value <0.05. Go analysis and KEGG analysis were performed to determine the biological significance of these differentially expressed genes.

### Statistics

Data represented as means ± standard deviations (SDs) unless otherwise specified. To determine statistical significance, Student’s *t*-tests and one-way analysis of variance (ANOVA) tests were used where indicated. A *P*-value of <0.05 was considered significant for all analyses.

## Clinical perspectives

- IGFBP7 has been found to play a crucial role in inflammatory diseases. However, the role of IGFBP7 in different phases of inflammatory diseases is unclear.
- In the present study, we found that IGFBP7 facilitates the repair of endothelial cells in the recovery phase rather than the acute phase of ALI, which may be related to the promotion of FOS phosphorylation and the up-regulation of YAP1 by IGFBP7.
- The present study indicates that IGFBP7 has diverse roles in different stages of ALI, expanding the understanding of IGFBP7 in ALI, and suggesting that IGFBP7 as a potential therapeutic target in ALI needs to take into account the period specificity of ALI.

## Supplementary Material

Supplementary Figures S1-S3 and Table S1

## Data Availability

Raw data of RNA-sequence were deposited into the NCBI database (GEO: PRJNA1022312). All data generated or analyzed during the present study are included in this article. Further inquiries can be directed to the corresponding author.

## Abbreviations

ALI: acute lung injury
AP-1: activator protein-1
ARDS: (acute respiratory distress syndrome
DAPI: 4′,6′-diamidino-2-phenylindole
DEG: differentially expressed gene
DMEM: Dulbecco’s Modified Eagle Medium
EDU: 5-Ethynyl-2'-deoxyuridine
IGFBP7: nsulin-like growth factor binding protein 7
YAP1: Yes-associated protein 1
HUVECs: Human Umbilical Vein Endothelial Cells
