# Supplementary material for: IGFBP7 promotes endothelial cell repair in the recovery phase of acute lung injury
Source: Clin Sci (Lond). 2024 Jun 21;138(13):797–815. doi: 10.1042/CS20240179 (PMC11196208; doi:10.1042/CS20240179)
Supplement: Supplementary Figures S1-S3 and Table S1 [file CS-2024-0179_supp.pdf]

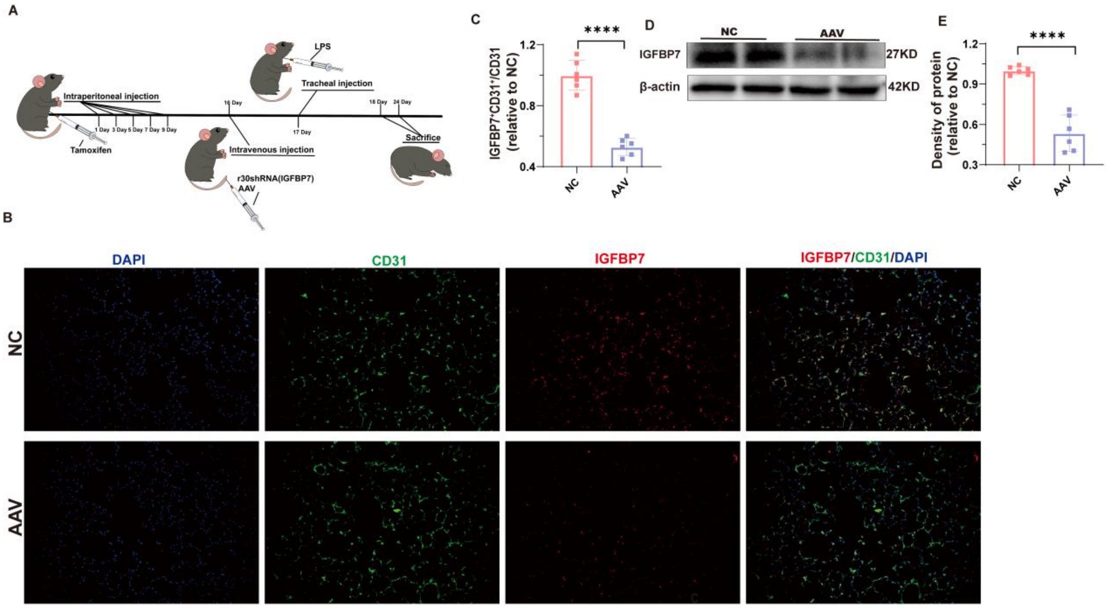

Figure S1. Validation of gene knock-down mice. (A) Tamoxifen was administered every other day for a total of five times, followed by a 7-day feeding period. Subsequently, AAVshRNA (shIGFBP7) was injected via the tail vein, and after 1 month, LPS (5 mg/kg) was administered via airway instillation. Mice were sacrificed after 1 day and 7 days of feeding to obtain specimens. **(B, C)** Co-staining of IGFBP7-AF555, CD31-AF488, and DAPI in lung tissues of normal and IGFBP7-cKD mice. Scale bar: 100 μm (20X). **(D, E)** Immunoblotting was performed to detect the expression of IGFBP7 protein in lung tissues of healthy and IGFBP7-cKD mice. β-Actin was used as an internal control for normalization. t-test (C, E). NC, Negative Control. AAV, Adeno-associated virus.

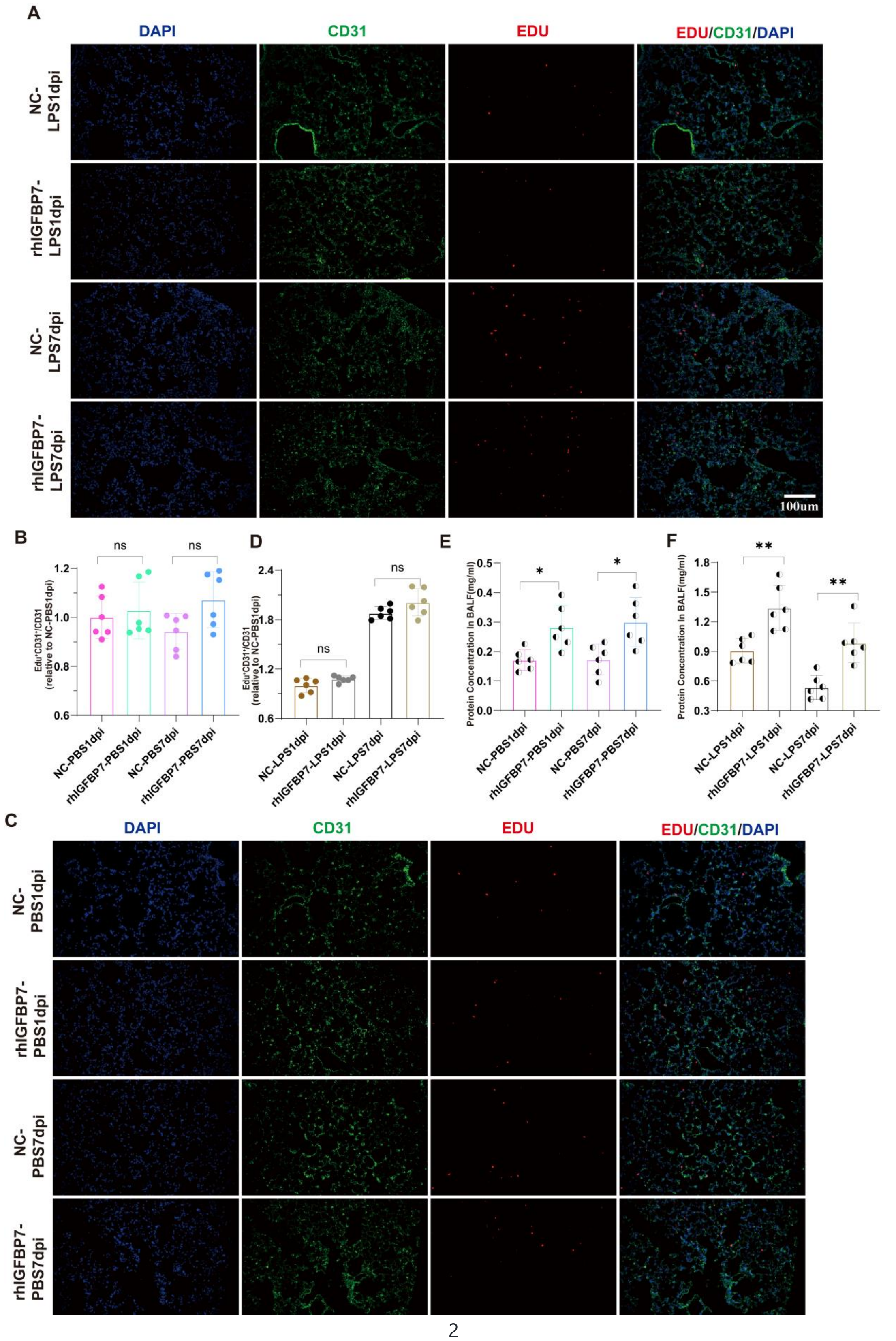

A
DAPI
CD31
EDU
EDU/CD31/DAPI
NC-LPS1dpi
rhIGFBP7-LPS1dpi
NC-LPS7dpi
rhIGFBP7-LPS7dpi
100um
B
ns
ns
Edu+CD31+/CD31 (relative to NC-PBS1dpi)
NC-PBS1dpi
rhIGFBP7-PBS1dpi
NC-PBS7dpi
rhIGFBP7-PBS7dpi
D
ns
ns
Edu+CD31+/CD31 (relative to NC-PBS1dpi)
NC-LPS1dpi
rhIGFBP7-LPS1dpi
NC-LPS7dpi
rhIGFBP7-LPS7dpi
E
Protein Concentration In BALF(mg/ml)
NC-PBS1dpi
rhIGFBP7-PBS1dpi
NC-PBS7dpi
rhIGFBP7-PBS7dpi
F
Protein Concentration In BALF(mg/ml)
NC-LPS1dpi
rhIGFBP7-LPS1dpi
NC-LPS7dpi
rhIGFBP7-LPS7dpi
C
DAPI
CD31
EDU
EDU/CD31/DAPI
NC-PBS1dpi
rhIGFBP7-PBS1dpi
NC-PBS7dpi
rhIGFBP7-PBS7dpi

Figure S2. Supplementation of rhIGFBP7 does not affect the proliferation of vascular endothelial cells in healthy and ALI mice. (A, B) Representative images of EDU-AF555, CD31-AF488, and DAPI co-staining in mouse lung tissues from mice intravenous injection of rhIGFBP7 and subjected to intratracheal injection with LPS (5mg/kg) treatment. Lung tissues were collected after 24 hours or 7 days. Scale bars 100 μm (20X). (C, D) Representative images of EDU-AF555, CD31-AF488, and DAPI co-staining in mouse lung tissues from mice intravenous injection of IGFBP7 and subjected to intratracheal injection with PBS treatment. Lung tissues were collected after 24 hours or 7 days. Scale bars 100 μm (20X). (E, F) Mice were intravenously injected with IGFBP7 and intratracheally instilled with PBS or LPS (5mg/kg), and bronchoalveolar lavage fluid was collected 24 hours or 7 days later to measure protein concentration. *P<0.05, **P<0.01 [one-way ANOVA, Tukey's test (B, D, E, F)]. NC, Negative Control. AAV, Adeno-associated virus.

Figure S3.

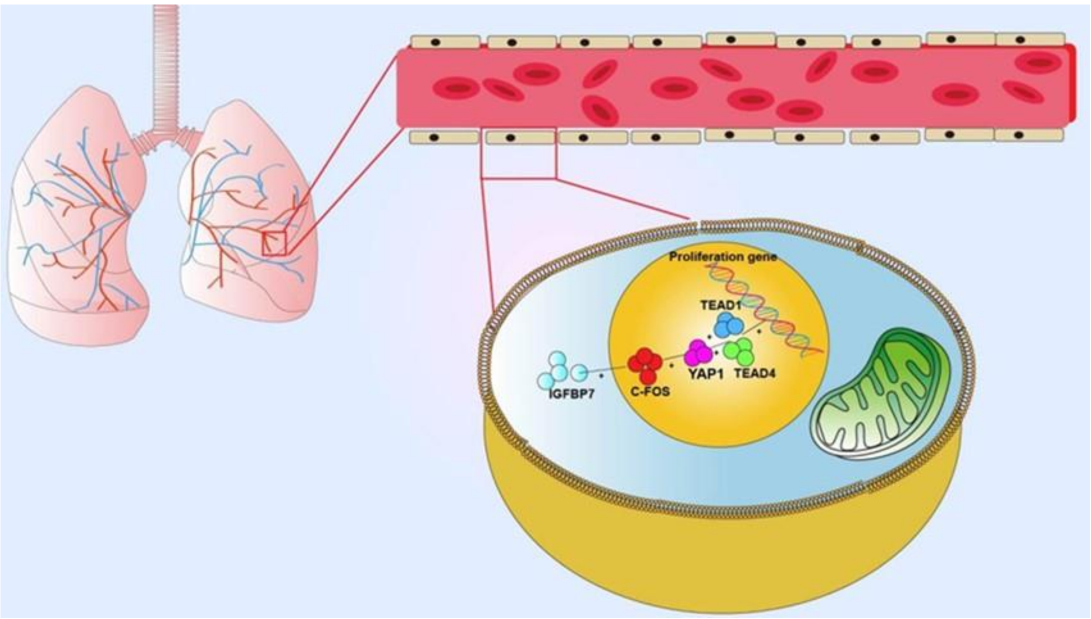

Table S1. List of specific primer sequences used for qRT-PCR

Species	Gene	Forward primer	Reverse primer
Human	CCNB1	AATAAGGCGAAGATCAACATGGC	TTTGTTACCAATGTCCCCAAGAG
Human	CCNC	CCTTGCATGGAGGATAGTGAATG	AAGGAGGATACAGTAGGCAAAGA
Human	CCND1	GCTGCGAAGTGGAAACCATC	CCTCCTTCTGCACACATTTGAA
Human	CCNE1	AGAGGAAGGCAAACGTGACC	TCAGTTTTGAGCTCCCCGTC
Human	KI67	TATCAAAAGGAGCGGGGTCG	GCTGGCTCCTGTTCACGTAT
Human	EGFR	AACTGTGAGGTGGTCCTTGG	GTTGAGGGCAATGAGGACAT
Human	SOX17	GACATGAAGGTGAAGGGCGA	GTTCAAATTCCGTGCGGTCC
Human	GAPDH	*CAAATTCCATGGCACCGTCA*	*GATGGCATGGACTGTGGTCA*
Mouse	Ccnb1	TAATCCCTCTCCAAGCCCGA	TTTGGGTCAGCCCCATCATC
Mouse	Ccnc	ACATGTGTGTTTTTGGCATCCA	GACCCTGCTCTCCTTCACTG
Mouse	Ccnd1	CCCTTGACTGCCGAGAAGTT	GGGGGTCCTTGTTTAGCCAG
Mouse	Ccne1	CTTATGGTGTCCTCGCTGCT	GGATAACCATGGCGAACGGA
Mouse	ki67	AGTCTCTTGGCACTCACAGC	ATTTTGTAGGGTCGGGCAGG
Mouse	Egfr	CAACATCCTGGAGGGGGAAC	CCACTGCCATTGAACGTACC
Mouse	Sox17	GATACGCCAGTGACGACCAG	CCCATGTGCGGAGACATCAG
Mouse	Gapdh	TCATCATCTCTGCCCCCTCT	GGGTGTCGCTGTTGAAGTCA
